# Local Solvent Ordering Drives Supramolecular Chirality Inversion

**DOI:** 10.1002/advs.76943

**Published:** 2026-08-03

**Authors:** Triza Pal, Akta Singh, Subinoy Adhikari, Jagannath Mondal, Debangshu Chaudhuri

**Affiliations:** ^1^ Department of Chemical Sciences Indian Institute of Science Education and Research (IISER) Kolkata India; ^2^ Tata Institute of Fundamental Research Hyderabad Telangana India

**Keywords:** chemistry, diimide, helicity, hydrogen  bond, molecular dynamic, perylene, solvation  shell, solvophobic, supramolecular chemistry

## Abstract

Solvent composition is widely assumed to influence supramolecular chirality indirectly through bulk properties such as polarity or solvophobicity. Here, we show that local solvent organization can directly dictate chiral pathway selection and invert supramolecular helicity. A chiral perylene bisimide bearing L‐phenylalanine substituents (**L‐PhePBI**) undergoes a complete reversal of helical sense in water/DMSO mixtures upon a narrow change in solvent composition (Δ*f*_water_ = 0.2), despite maintaining a similar degree of aggregation. The comparable polarity of the two solvent compositions excludes bulk solvent effects as the origin of this stereomutation. Molecular dynamics simulations reveal that solvent‐composition‐dependent reorganization of the first hydration shell biases molecular twist angles within stacked assemblies, thereby selecting opposite chiral pathways. Variable‐temperature spectroscopic studies further identify a kinetically trapped chiral state that irreversibly converts to a thermodynamically stable enantiomorph upon heating. This thermal inversion is associated with a redistribution of hydrogen bonding from nearest‐neighbor to nonadjacent molecular pairs, stabilizing a distinct packing motif. Together, these results establish a direct mechanistic link between local solvent ordering, noncovalent interaction reorganization, and supramolecular stereomutation, demonstrating solvent composition as a precise control parameter for programming chiral organization in supramolecular materials.

## Graphical abstract

Local solvent organization is shown to govern supramolecular chirality by selecting distinct chiral assembly pathways. A subtle change in water‐DMSO composition reverses the helical sense of a chiral perylene bisimide, while heating induces an irreversible thermal inversion to a more stable enantiomorph. The findings establish local solvation as a precise handle for programming supramolecular chirality.

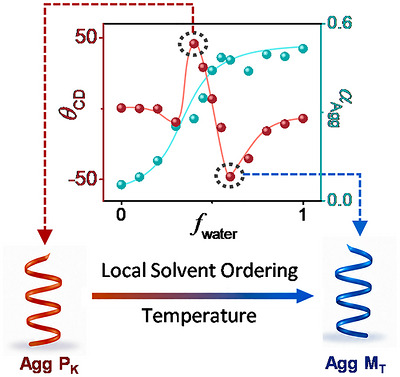

## 1 Introduction

Chirality in natural world is ubiquitous and transcends dimensions, from molecular to cosmological length scales [1]. From the chemist's viewpoint, the interest in molecular chirality stems from the fact that biological systems are often homochiral, and several important biological functions depend on enantiospecific interactions and binding [2, 3]. Chirality at mesoscopic length scales, on the other hand, bridges the molecular and the macroscopic world, giving rise to unique materials that can harness properties such as spin‐polarized transport [4] and other chiroptical and magneto‐chiral [5] phenomena. Molecular self‐assembly offers a convenient and reliable way of creating chirality at mesoscopic length scales [6, 7, 8].

Achieving homochirality in a supramolecular structure requires that the self‐assembly be carried out under a clear chiral bias [9, 10, 11]. Such bias is most commonly provided by the chirality of the molecular building block, which ensures that a pair of enantiomers will assemble under identical conditions into enantiomorphic structures [9]. More intriguing examples of such chiral bias include the well‐known majority‐rules effect [12, 13], where a tiny excess of one enantiomer in a nearly‐racemic mixture can impose homochirality to the overall assembly. Alternatively, a small number of chiral “sergeant” molecules can force a large excess of achiral “soldiers” into a cooperatively assembled homochiral structure [14, 15]. The chiral bias can also be introduced through external factors, like solvent [16, 17, 18], mechanical [19, 20] (stirring or vortex mixing), and optical (circularly polarized light) stimuli [21, 22] of a specific handedness. Being ubiquitous in all self‐assembly processes, solvents play a pivotal role in self‐assembly [23, 24, 25]. Understanding the influence of solvent‐solute interactions on supramolecular chirality can pave the way to control and modulate chirality in a variety of assembled materials [16, 26]. There are different ways in which a solvent can influence supramolecular chirality. In a chiral solvent, achiral monomers can assemble into homochiral structures by exploiting small differences in the solvation energy (preferential chiral solvation) [27, 28, 29]. This effect is particularly pronounced in cooperatively bound assemblies, where preferential chiral solvation can even override the helical preference of a chiral monomer [30]. A solvent can also influence supramolecular chirality through co‐assembly, as was demonstrated in the case of coronene bisimide self‐assembly [31], wherein intercalation of different (achiral) alkane solvent molecules into the assembled structure could cause a helix reversal, under thermodynamic control [32]. Likewise, empirical correlations between supramolecular chirality and solvent polarity or micro‐heterogeneity of solvent mixtures have also been reported [16, 17, 33]. It is worth noting that solvent‐dependent supramolecular chirality inversion can also occur under kinetic control [11], whereby a less stable kinetic enantiomorph transforms into the more stable thermodynamic state with opposite chirality [34]. Nevertheless, how an achiral solvent might modulate the chiral bias remains an interesting and relevant question even in the context of supramolecular stereomutation under kinetic control.

We recently reported an unusual solvent‐composition dependence of the supramolecular chirality of a chiral perylene bisimide (PBI) molecule. **D‐PhePBI** [35]. In water‐cosolvent (DMSO/dioxane) mixture, the molecule assembled into weakly chiral nanospheres. But, in a very narrow solvent composition range, these nanospheres are spontaneously converted to strongly chiral nanowires, a transformation mediated by secondary nucleation. More intriguingly, the supramolecular chirality of the nanowires could be completely reversed by changing the cosolvent from DMSO to dioxane. Clearly, the only source of chirality is the molecule itself, and yet changing the achiral cosolvent could alter the final outcome so drastically. Evidently, the origin of chiral bias in these binary solvent mixtures cannot be explained within the established frameworks (polarity and micro‐heterogeneity), which prompts the need for further investigations into the origin of this solvent‐dependent supramolecular chirality.

Herein, we present a combined experimental and computational investigation into the unusual reversal of supramolecular chirality of **L‐PhePBI** assemblies in water‐DMSO mixtures. Contrary to expectation, the solvent composition dependence of supramolecular chirality of the L‐isomer does not completely mirror the assembly characteristics of the D‐isomer. A small change in the solvent composition drives a complete inversion of supramolecular chirality, highlighting the fact that the chiral bias for self‐assembly is not defined solely by the point chirality of the molecule; its interaction with the solvent medium does play a crucial part. Interestingly, the chirality of one of the assembled states can also be reversed thermally, indicating the possibility of a kinetic effect. We carried out a detailed molecular dynamics simulation to show how the change in the local hydration structure is responsible for the observed solvent‐composition‐dependent stereomutation. Thermal switching of supramolecular chirality, on the other hand, is driven by an unusual reordering of the nearest and distant H‐bonding interactions. This work not only provides a fundamental insight into the emergence of supramolecular chirality but also establishes solvent engineering as a powerful tool for controlling the chiral organization of molecules in the assembled state.

## 2 Results and Discussion

**FIGURE 1:**
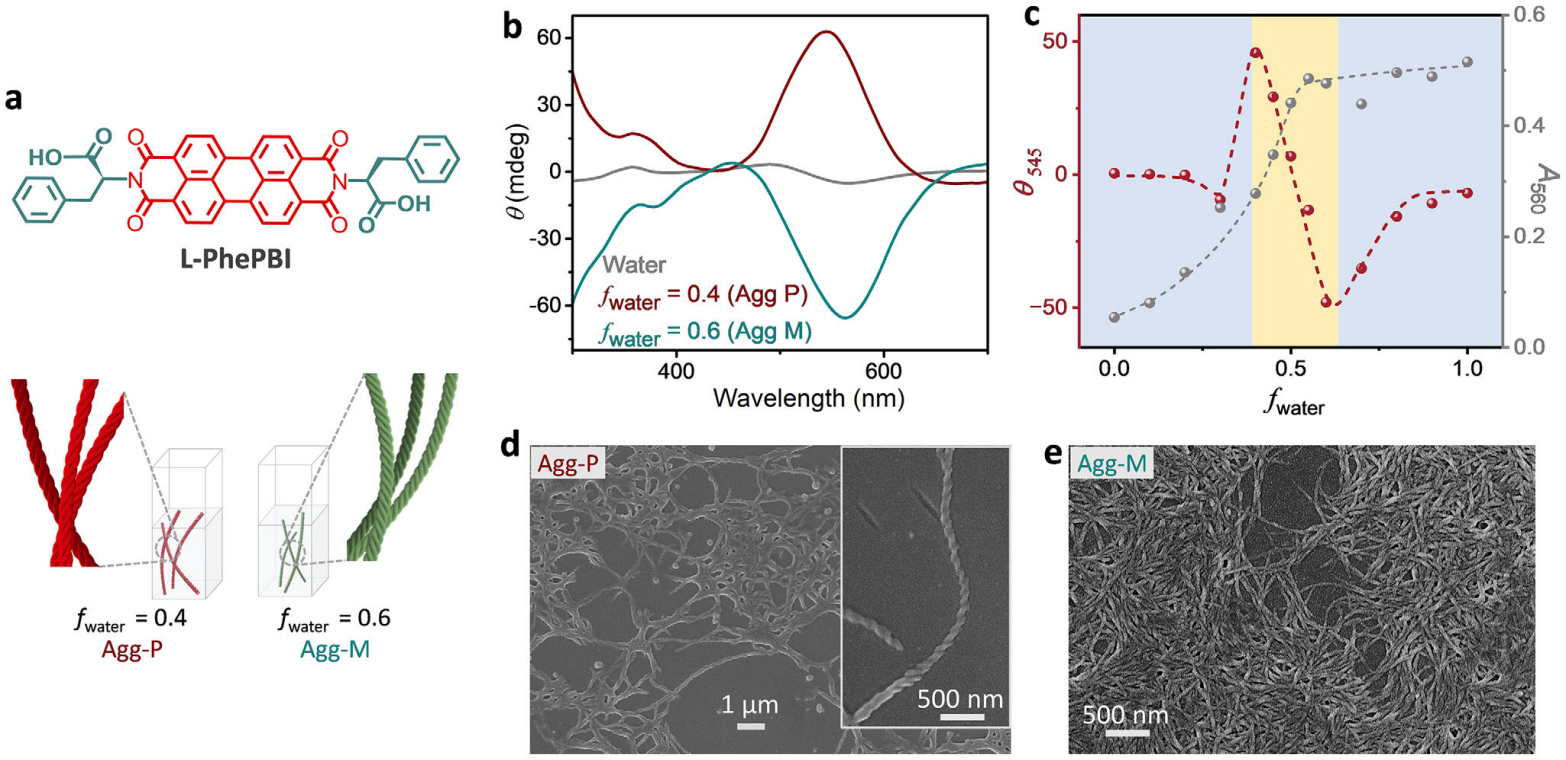
Solvent‐composition dependent supramolecular chirality. (a) The molecular structure of **L‐PhePBI** shows the central PBI unit (in red) and L‐Phe at the imide position. The schematic shows contrasting supramolecular chirality in two DMSO‐water mixtures. (b) CD spectra of self‐assembled **L‐PhePBI** in *f*_water_ = 0.4 and 0.6 DMSO‐water mixtures feature opposite chiralities. (c) Composition‐dependent changes in absorbance at 560 nm (*A*_560_) and CD intensity at 545 nm (*θ*_545_) monitor the degree of aggregation and emergence of supramolecular chirality. Dashed lines are guide to eye. Contrasting FESEM morphologies of **L‐PhePBI** supramolecular polymers in (d) *f*_water_ = 0.4 DMSO–water; the inset shows a prominently clockwise helical twist (e) *f*_water_ = 0.6 composition; conc: 300 µM, 298 K.

The self‐assembly behavior of **L‐PhePBI**, a chiral PBI symmetrically substituted with L‐phenylalanine residues at the imide positions (Figure 1a), was investigated in water–DMSO mixtures. At a volume fraction of water (*f*_water_) below 0.4, the degree of aggregation of **L‐PhePBI** remains very low. The resultant circular dichroism (CD) spectrum features a very weak signal intensity in the range of 400−650 nm (Figure S3a). At *f*_water_ = 0.4 (60% v/v DMSO in water) composition, appearance of a strong positive dichroic signal at 545 nm (see Figure 1b) marks the formation of a homochiral assembly (Agg‐P). Incidentally, in the most aggregating medium, that is, pure water, supramolecular chirality of **L‐PhePBI** is very poorly expressed, featuring a weakly negative bisignate Cotton effect in pure water (Figure 1b and Figure S3a). We note that the assembly characteristics of **L‐PhePBI** in *f*_water_ = 0.4 and 1 DMSO‐water solutions are, as expected, complementary to those of **D‐PhePBI** in *f*_water_ ≈ 0.4 and 1 solution [35]. However, the notion that opposite enantiomers under identical conditions assemble into enantiomorphic structures is contradicted in *f*_water_ = 0.6 DMSO‐water solution. For a small change in the solution composition from *f*_water_ = 0.4 to 0.6, we observe a complete inversion of the CD signal from a positive to a negative Cotton effect (see Figure 1b), which indicates a complete reversal of supramolecular chirality to Agg‐M. Incidentally, this drastic change in the supramolecular chirality does not drastically alter the corresponding absorption spectra (Figure S3b), underscoring the fact that while a change in solvent composition modulates the supramolecular chirality, the degree of **L‐PhePBI** aggregation remains quite similar in both cases. The composition‐dependence of CD intensity at 545 nm (*θ*_545_) (Figure 1c) captures the sharp and unusual dependence of supramolecular chirality on the solvent‐composition. In contrast, the plot of absorbance at 560 nm (*A*_560_) reveals a simple monotonic transition from molecularly dissolved **L‐PhePBI** to H‐aggregated species. Thus, although the overall aggregation process appears straightforward, the resulting supramolecular chirality displays a far more intricate dependence on solvent composition, underscoring the critical role of solvent–solute interactions in defining the chiral bias under which the self‐assembly proceeds. Figure 1d–e illustrate the distinct morphologies of **L‐PhePBI** assemblies formed in DMSO–water mixtures. Both Agg‐P and Agg‐M exhibit predominantly fibrillar morphologies. Notably, Agg‐P features right‐handed helically twisted fibrils (Figure 1d), with the inset highlighting the characteristic clockwise twist. In contrast, the helical twist is not as clearly discernible in Agg‐M (Figure 1e). In pure water, **L‐PhePBI** assemble into spherical nanostructures, as shown in Figure S3c (Conc. 300 µM). It is worth noting that the empirical solvent polarity parameter, *E*_T_(30), for the two solvent compositions are nearly identical (Figure S3d) [36], which rules out bulk solvent polarity as the driving force for the observed differences in supramolecular chirality. Further to check the generality of this behavior, we explored **L‐PhePBI** self‐assembly in other water‐cosolvent binary mixtures, over the entire composition range. The cosolvents used were 1,2‐dioxane, dimethylformamide (DMF), and acetonitrile. In each of these binary solvent mixtures, **L‐PhePBI** organizes into a chiral assembly above *f*_water_ = 0.3 compositions, evident from the appearance of a negative Cotton effect in the CD spectrum (Figure S4) that is quite similar to that of Agg‐M in *f*_water_ = 0.6 DMSO‐water mixture. However, no composition dependent chirality inversion was observed in these binary solvent mixtures.

**FIGURE 2:**
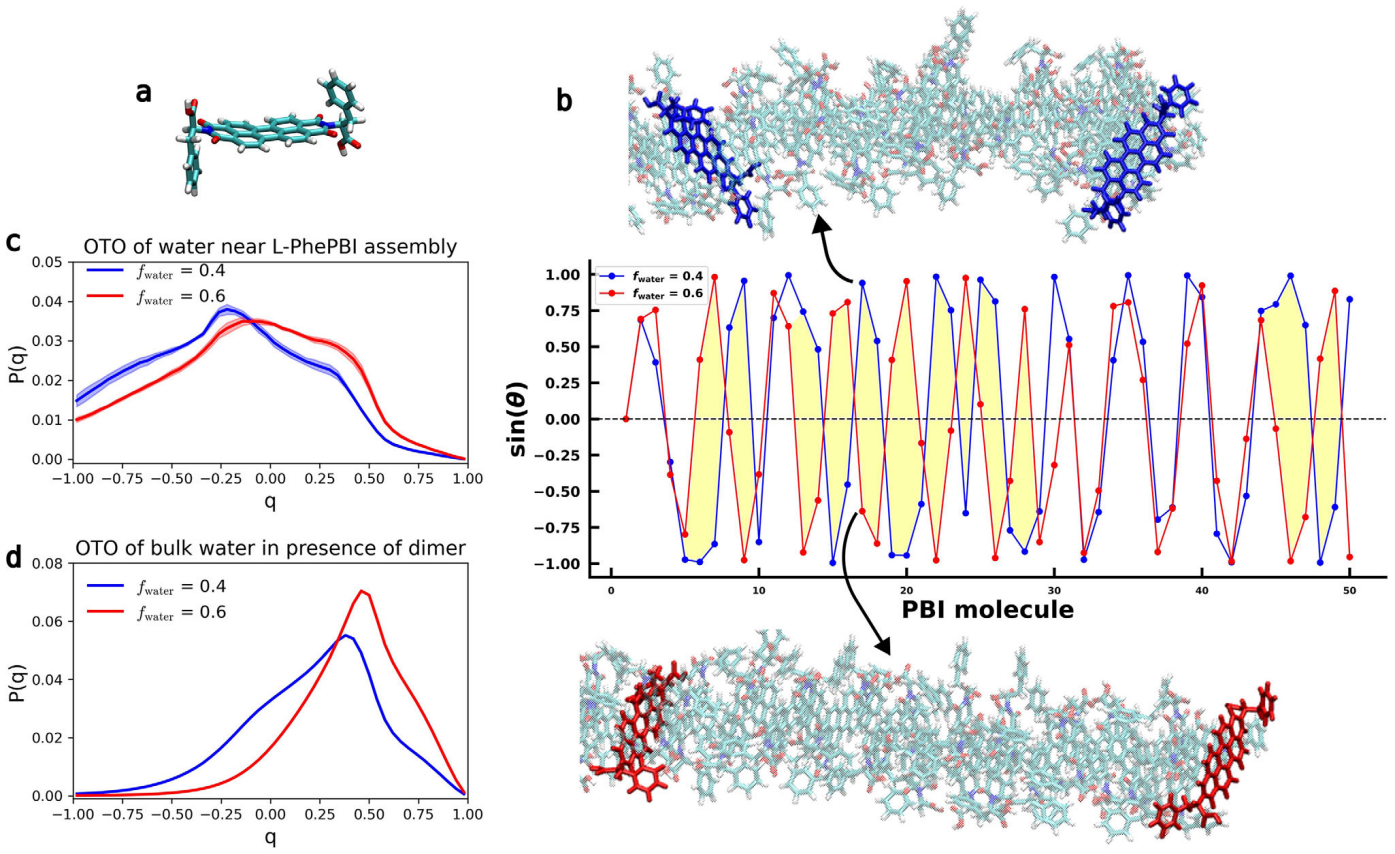
MD simulation of solvent‐composition dependent supramolecular chirality. (a) Model of **L‐PhePBI** molecule. (b) Median plot of the sine of twist angles of assembly molecules relative to the first molecule, measured during the simulation. Representative snapshots highlight instances where molecules exhibit opposite twist angles. (c) Probability density distribution of the orientational tetrahedral order parameter (OTO) for water within 0.5 nm of the **L‐PhePBI** assembly (averaged over four randomly selected molecules). (d) Probability density distribution of the OTO parameter for bulk water.

To gain a molecular‐level insight into the origin of the observed chiral inversion in water‐DMSO mixtures, molecular dynamics (MD) simulations were performed. At the beginning of the simulation, **L‐PhePBI** molecules (Figure 2a) were randomly inserted in the solvent boxes. In neat DMSO, the molecules remain dispersed, indicating that **L‐PhePBI** is molecularly dissolved in DMSO (Figure S5). In pure water, **L‐PhePBI** molecules form a disordered aggregate (Figure S6a). Interestingly, in the two binary solvent mixtures, the monomers assemble into stacked aggregates (Figure S6b and c). Twist angle analysis of the nearest neighbors in the stacked assemblies gives a first peak at ≈ 40° angle (see method section in Supporting Information). Hence, to further investigate, we place the preformed assembly of 50 **L‐PhePBI** molecules oriented at ∼ 40° twist angle between nearest neighbors into *f*_water_ = 0.4 and 0.6 water‐DMSO mixtures (details mentioned in Supporting Information). The assemblies remain intact even after a 100 ns simulation in both solvent mixtures (Figure S7a, b). Analysis of twist angles, measured relative to the bottom‐most molecule, shows that although both systems start from the same configuration (Figure S5d), the binary solvents induce opposite orientational preferences in parts of the stack (Figure 2b). To further compare the molecular packing in the two systems, molecules exhibiting large orientation differences (|Δsin(θ)| ≥ 0.5) were identified and their orientational profiles were analyzed. These profiles were found to be strongly anticorrelated (Pearson correlation coefficient, *r* = −0.863), indicating that molecules preferentially adopt the opposite orientation in the two solvent compositions.  Fourier analysis of the orientational time series further supports this inversion in molecular ordering (Figure S8), with the averaged spectra of the selected molecules exhibiting opposite signs for the two solvent compositions. These results indicate that the binary solvents subtly but decisively influence the orientational preferences of **L‐PhePBI** molecules, thereby providing a molecular basis for the observed chiral inversion.

To characterize the local water structure, we evaluated the orientational tetrahedral order (OTO) parameter for water molecules within 0.5 nm of the stacked **L‐PhePBI** assembly. This cutoff length scale corresponds to the first hydration shell of water molecules, as determined from the radial distribution function (Figure S9). We find that water exhibits slightly different orientational distributions in the two solvent compositions. To confirm that this result is independent of the **L‐PhePBI** molecules’ position in the assembly, we randomly select four molecules and calculate the OTO parameter of water around each (Figure 2c and Figure S10). The resulting plots show that in mixtures with higher water content, water adopts a more ordered structure around the assembly compared to mixtures with lower water fractions. This difference arises because at higher DMSO fractions, DMSO disrupts the ordering of water molecules [37]. To compare with bulk water, and given the computational cost of evaluating the OTO parameter in large systems, we study the case of **L‐PhePBI** dimer. The probability density distribution of OTO parameter values for water within the first hydration shell closely resembles that of the stacked assembly (Figure 2c and Figure S11). Calculating the OTO parameter for bulk water reveals a similar trend to that observed near the dimer (Figure 2d). To further probe solvent‐solute interactions, we analyzed the continuous hydrogen‐bond lifetime between water and **L‐PhePBI** (Figure S12). The average hydrogen‐bond lifetimes were found to be more than an order of magnitude longer than those typically reported for bulk water [38], indicating slower hydrogen‐bond dynamics in the vicinity of **L‐PhePBI**. Moreover, the hydrogen‐bond lifetime decreases with increasing water fraction, suggesting that the local hydration environment becomes more dynamic at higher water content. Interestingly, the solvent composition exhibiting a lower OTO value shows a longer **L‐PhePBI**‐water hydrogen‐bond lifetime, whereas the composition with a higher OTO value exhibits a shorter lifetime. Since OTO characterizes water‐water tetrahedral ordering and hydrogen‐bond lifetime reflects the persistence of direct solute‐water interactions, these analyses suggest a connection between the organization of the water network and the stability of solute‐water hydrogen bonds modulated by solvent composition. These results suggest that variations in solvent composition modulate the ordering of water and that the local solvent arrangement around aggregating molecules plays a decisive role in controlling aggregate symmetry. Even subtle shifts in composition can reorganize the solvation environment, thereby exerting a profound influence on the emergence of supramolecular chirality. This finding highlights the critical importance of solvent–solute interactions in pathway selection and chiral inversion.

**FIGURE 3:**
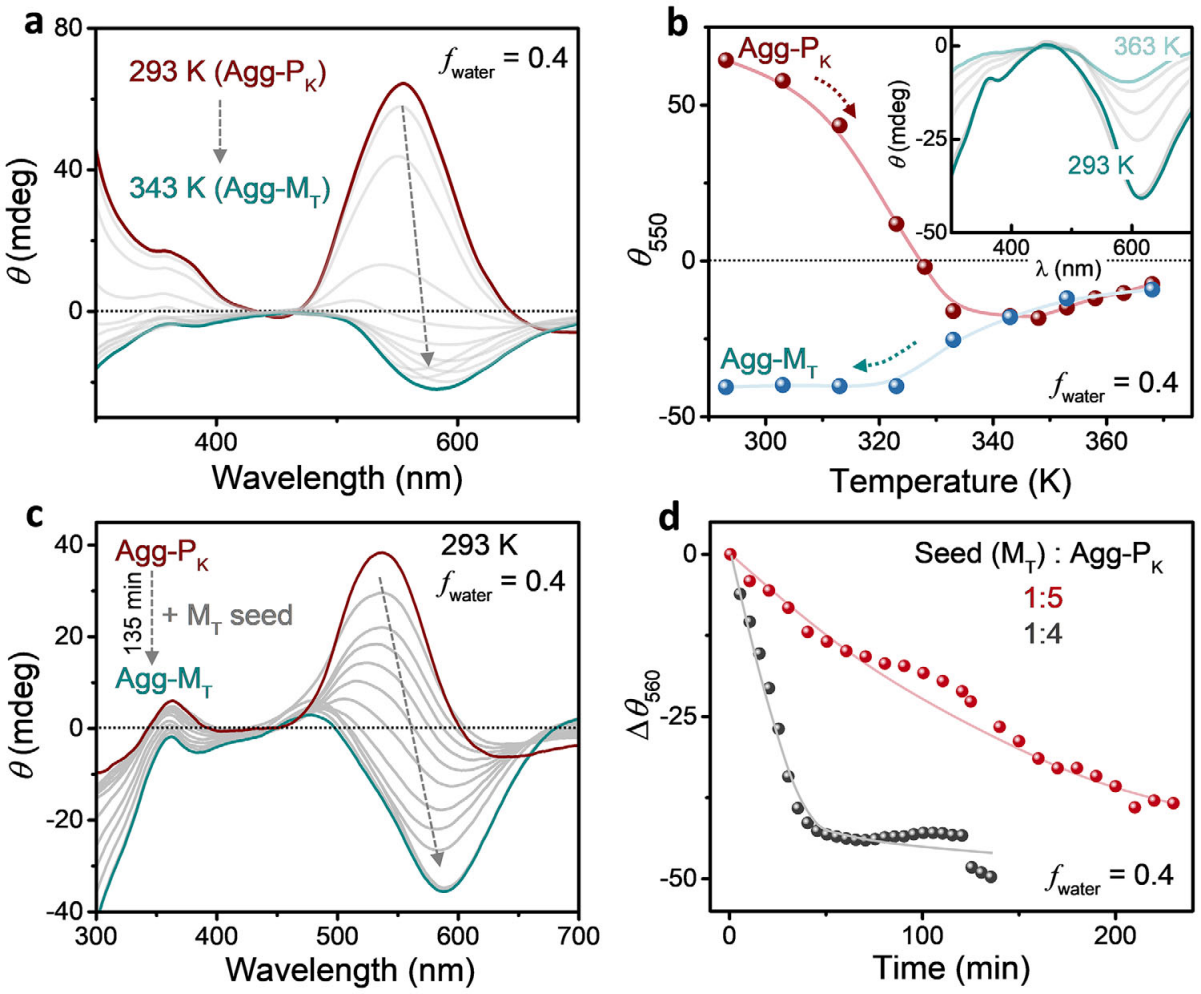
Thermal stereomutation of **L‐PhePBI** assembly in *f*_water_ = 0.4 solution, conc. 300 µM. (a) Upon heating (1°C/min), the CD spectrum gradually changes from a positive cotton effect at 293 K (Agg‐P_K_) to a negative cotton effect at 343 K (Agg‐M_T_). (b) Temperature dependence of CD intensity at 550 nm (*θ*_550_) shows an irreversible Agg‐P_K_ → Agg‐M_T_ transformation. Solid lines are guide to eye. The inset shows the growth of Agg‐M_T_ during the cooling run. (c) Seed‐induced inversion of Agg‐P_K_ to Agg‐M_T_, at 293 K. (d) Kinetics of Agg‐P_K_ → Agg‐M_T_ stereomutation at 293 K, for two different seed−to−Agg‐P_K_ ratios. A faster kinetics is observed at greater seed concentrations, total conc. 300 µM.

The formation mechanisms of the two enantiomorphic assemblies (Agg‐P and Agg‐M) were investigated using variable‐temperature (VT) absorption and CD spectroscopy in *f*_water_ = 0.4 and 0.6 DMSO–water mixtures. At *f*_water_ = 0.6, VT‐CD measurements (Figure S13a, b) reveal a gradual decrease in the CD intensity upon slow heating (at 1 K min^−1^) from 298 to 363 K, indicative of a partial loss of supramolecular chirality. However, a complete disassembly is not observed. Upon slow cooling, the CD intensity fully recovers, accompanied by a ≈20 nm red shift, which could be attributed to the formation of larger aggregates (Figure S13a, b). The observed thermal reversibility indicates that in *f*_water_ = 0.6 mixture, assembly of **L‐PhePBI** into Agg‐M happens under thermodynamic control (Figure S13a–d). In contrast, Agg‐P in *f*_water_ = 0.4 exhibits a thermally induced chirality inversion (Figure 3a). At 293 K, the CD spectrum displays a positive Cotton effect centered at 550 nm, characteristic of the as‐prepared aggregate. Upon slow heating (1 K min^−1^), the CD signal starts to diminish and undergoes a complete inversion into a negative bisignate Cotton effect at 343 K. Notably, upon slow cooling, the inverted CD signature is retained and shows a slight enhancement in chiral asymmetry (inset to Figure 3b), indicating an irreversible thermal supramolecular stereomutation from P‐type to M‐type assembly. The temperature dependence of the CD intensity at 550 nm (*θ*_550_) in *f*_water_ = 0.4 solution, presented in Figure 3b, summarizes the irreversible thermal stereomutation. A greater stability of the assembly formed after thermal cycling is evident from the fact that a subsequent thermal cycling from 293 to 363 K and back does not cause any further assembly reorganization (Figure S14). We tentatively denote the two enantiomorphic aggregates in *f*_water_ = 0.4 as Agg‐P_K_ (the as‐prepared kinetically trapped state) and Agg‐M_T_ (the thermodynamically stable state formed after thermal cycling). VT absorption spectra further support this conclusion (Figure S15). The as‐prepared Agg‐P_K_ displays a weaker H‐type excitonic coupling. Thermal cycling results in a decrease in the O.D. and appearance of a new band around 610 nm that indicates an improved H‐type coupling and consequently a hypochromism in Agg‐M_T_. To further verify the kinetic nature of Agg‐P_K_, we investigated the effect of heating and cooling rates on the thermal stereomutation process. A faster rate (5 K min^−^^1^) resulted in the recovery of the original Agg‐P_K_ state (Figure S16), demonstrating faster heating and cooling can trap the kinetic (Agg‐P_K_) state, and prevent its reorganization into the more stable Agg‐M_T_ state [34, 39]. Field‐emission scanning electron microscopy (FESEM) images of Agg‐M_T_ (Figure S17a) reveal the presence of helical fibers, with apparently a much lower screw pitch than Agg‐P_K_, thus consistent with a significant reorganization of molecular packing. Finally, linear dichroism (LD) measurements confirm that LD contributions are negligible for both aggregates (Figure S17b), verifying that the observed CD spectral features represent genuine chiroptical response rather than orientational artifacts.

A greater stability of Agg‐M_T_ over Agg‐P_K_ allowed us to perform a seeding‐induced chiral inversion. Seeds of Agg‐M_T_ were added to a solution of as‐prepared Agg‐P_K_ at 298 K (see SI for details), and the evolution of the assembled state was monitored using CD spectroscopy (Figure 3c). Upon the addition of seed, Agg‐P_K_ undergoes a progressive transformation into Agg‐M_T_, even at 293 K. The kinetics of Agg‐P_K_ → Agg‐M_T_ conversion correlates directly with the amount of Agg‐M_T_ seed added, as shown in Figure 3d. Notably, in the absence of either thermal activation or seed addition, Agg‐P_K_ remains kinetically stable for one week (Figure S18), highlighting the metastable nature of this assembly. Importantly, Agg‐M_T_ formed either through thermal annealing or via seed‐induced conversion remains unchanged upon subsequent thermal cycling, confirming that Agg‐M_T_ represents the thermodynamically stable aggregate of **L‐PhePBI** in the *f*_water_ = 0.4 DMSO–water mixture.

Simulations with different initial configurations were performed at varying temperatures to further assess the origin of the temperature‐induced chiral inversion. At 298 K, the assemblies remained intact irrespective of the starting configuration (Figure S19a, b). In contrast, at 363 K, most assemblies begin to break into smaller fragments (Figure S19c).  We expect that an increase in temperature will give rise to differences in the assembly structure. To verify this, we calculate the twist angle between neighbouring molecules in the assembly and plot its probability density distribution (Figure S20a) (see supporting information for details). At 298 K, the probability peaks at about −40° and +40° angles. However, at 363 K, the probability of these angles decreases with a more prominent contribution from larger angles around ±70° to ±80°. This is observed because at high temperatures, more states become accessible to the assembly system. The associated free energy profile, obtained from Δ*G*  =   − *RT*ln *P*/*P_max_*, where *P* represents the probability density, corroborates this behavior (Figure S20b).

**FIGURE 4:**
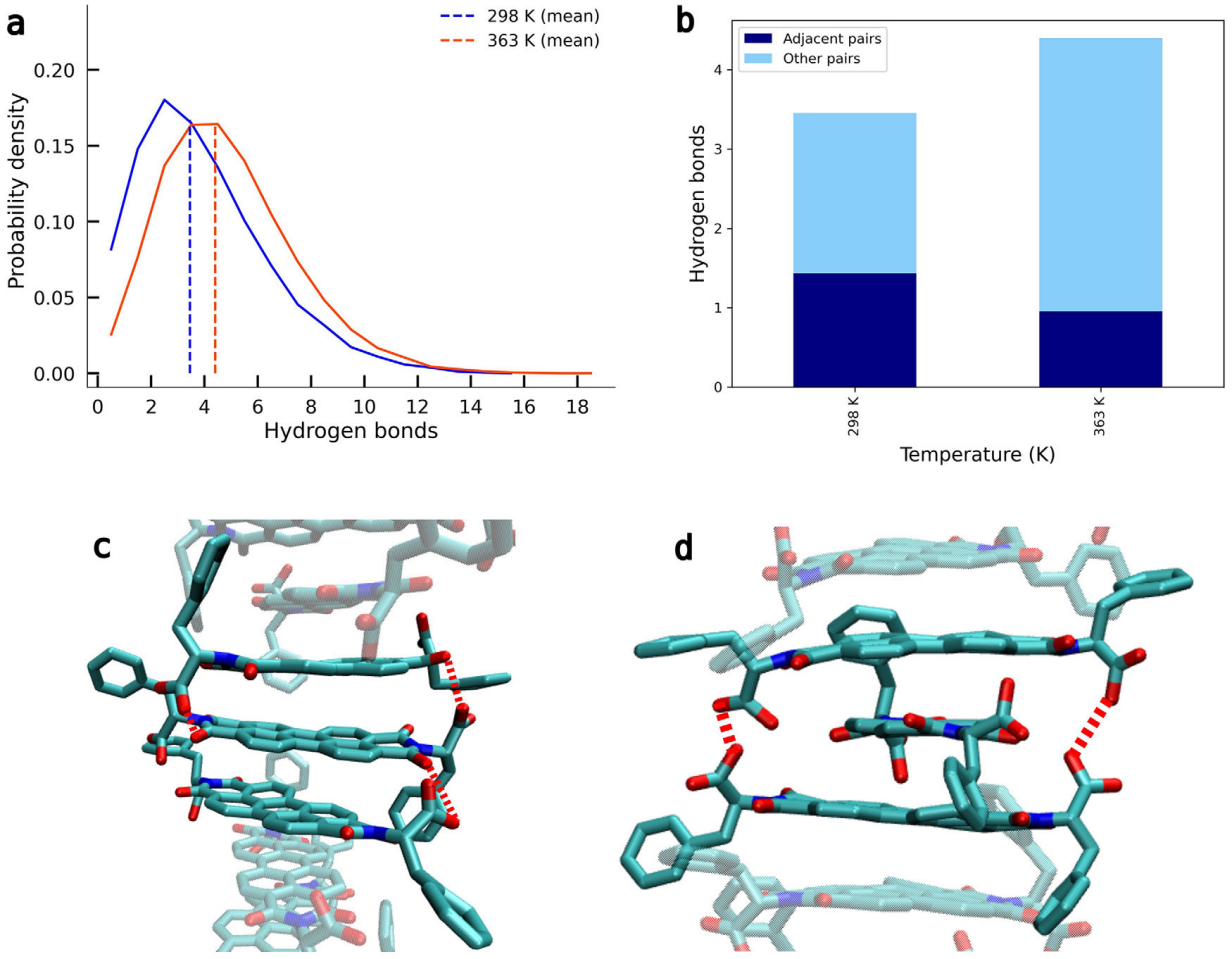
Understanding thermal stereomutation. (a) Total hydrogen bonds per frame in the entire assembly and (b) hydrogen bond contribution from adjacent pairs and other (non‐adjacent) pairs in an assembly per frame at 298 and 363 K. Representative snapshots of (c) hydrogen bonding between adjacent pairs and (d) hydrogen bonding between other pairs. Hydrogen bonds are depicted as red dashed lines. Water and DMSO molecules are not shown for clear visualization.

We calculate the number of hydrogen bonds per frame using a 0.35 nm cutoff distance between donor–acceptor pairs and a 150° cutoff angle for the donor–hydrogen–acceptor. For all initial configurations, we plot the probability density distribution of hydrogen bonds at both temperatures (Figure 4a). On average, the assembly at 363 K forms more hydrogen bonds than at 298 K, indicating that higher temperature facilitates the formation of new bonds. We further calculate the contribution of hydrogen bonds between adjacent molecules and non‐adjacent molecules (Figure 4b). At 363 K, the contribution from adjacent pairs decreases, while that from distant pairs increases, leading to an overall rise in the number of hydrogen bonds. This effect can be attributed to an increased distortion of the 1D assembly at higher temperatures. The increase in twist angles between adjacent molecules lowers the probability of nearest‐neighbor hydrogen bonding [37], which in turn leads to interaction with non‐adjacent molecules. Figure 4c and d include representative snapshots of hydrogen bonding between adjacent and non‐adjacent pairs. Together, these results suggest that higher temperatures facilitate structural rearrangements by reducing nearest‐neighbor hydrogen bonding and enhancing non‐adjacent interactions. The resulting reorganization of the hydrogen‐bond network provides a plausible molecular origin for the observed chiral inversion and may contribute to the thermodynamic preference of the negatively twisted assembly at elevated temperature. This reorganization not only stabilizes the assemblies but also provides a plausible molecular origin for the observed chiral inversion.

The interactions between the solvent and solute play a vital role in supramolecular assemblies, influencing the stability and properties of the resulting aggregates. But to understand the exact mechanism that is responsible and the type of solvent‐solute interaction involved in the observed behavior is still very challenging. In our previous work, we conjectured that the solvent‐solute interaction may be partially accountable for different chiral structure formations in different solvent compositions. Yet, the molecular basis for the contrasting supramolecular architectures observed across solvent systems—particularly in assemblies stabilized by multiple weak non‐covalent interactions—remained unclear. In the present study, the combination of experimental observations and theoretical analysis provides a comprehensive understanding of how solvent–solute interactions dictate pathway selection, promote chiral structure formation, and enable supramolecular chirality inversion.

## 3 Conclusions

In summary, our findings highlight the decisive role of solvent composition in directing supramolecular chirality and pathway selection in **L‐PhePBI** assemblies. A narrow variation in the water fraction of DMSO–water mixtures induces a complete inversion of helical sense, demonstrating how subtle changes in the local solvent environment can drastically alter chiral outcomes without significant changes in aggregation extent, reinforcing the notion that supramolecular chirality can be decoupled from conventional aggregation metrics. As observed previously for D‐PhePBI, this sharp solvent‐composition dependence points to a singularity in solvent–solute interactions rather than a gradual change in bulk solvent properties.

By integrating spectroscopic, microscopic, and molecular dynamics analyses, we identify solvent‐dependent restructuring of the local hydration environment as the key mechanistic driver of stereomutation, which biases molecular twist preferences and directs assembly along distinct chiral pathways. Thermal and seed‐induced experiments further reveal a clear kinetic–thermodynamic hierarchy, wherein a metastable chiral state undergoes irreversible conversion to a thermodynamically favored enantiomorph through temperature‐driven redistribution of hydrogen‐bonding interactions.

Together, these results establish a unified framework in which local solvation, pathway complexity, and kinetic trapping collectively dictate supramolecular chirality. More broadly, this work suggests practical design rules for chiral supramolecular materials: controlling solvent composition near critical regimes can be used to access, invert, and stabilize distinct chiral states without altering molecular structure, providing a powerful strategy for solvent‐engineered chiroptical and adaptive functional systems.

## Author Contributions

**T.P**. synthesis of the molecules, spectroscopic and microscopy studies. **A.S**., **S.A**., and **J.M**. theoretical calculations. **D.C**. conceptualization, supervision. The manuscript was written through the contributions of all authors. All authors have approved the final version of the manuscript.

## Conflicts of Interest

The authors declare no conflicts of interest.

## Supporting information




**Supporting File**: advs76943‐sup‐0001‐SuppMat.docx.

## Data Availability

The data that supports the findings of this study are available in the supplementary material of this article.
