# Supplementary material for: Local Solvent Ordering Drives Supramolecular Chirality Inversion
Source: Adv Sci (Weinh). 2026 Aug 3:e76943. Online ahead of print. doi: 10.1002/advs.76943 (PMC13430924; doi:10.1002/advs.76943)
Supplement: Supplementary file 1 — Supporting File: advs76943‐sup‐0001‐SuppMat.docx. [file ADVS-9999-e76943-s001.docx]

Supporting Information

Local Solvent Ordering Drives Supramolecular Chirality Inversion

*Triza Pal ^1^, Akta Singh ^2^, Subinoy Adhikari ^2^, Jagannath Mondal*,^2^ Debangshu Chaudhuri ^1^**

^1^ Department of Chemical Sciences, Indian Institute of Science Education and Research (IISER) Kolkata, Madhapur 741246, India.

^2^ Tata Institute of Fundamental Research, Hyderabad, Telangana, 500046, India

**Table of Contents:**

**1. Materials and Methods**

**2. Syntheses and Characterization**

**3. Additional Information**

**4. References**

**1. Materials and Methods:**

All reagents were purchased from Sigma Aldrich Chemical Co. and used without further purification. Spectroscopic-grade solvents were used for photophysical studies.

**NMR** spectra were recorded on AVANCE III 500 BRUKER spectrometer, and the data were

calibrated against TMS.

**HRMS** were recorded on Waters xevo G2-XS HD high-resolution Q-TOF LC-MS/MS benchtop

Spectrometer and Bruker Maxis Impact HD high-resolution Q-TOF LC-MS/MS benchtop

spectrometer.

**UV/Visible absorption spectroscopy** was performed on a Shimadzu UV-2600 spectrometer, single cell holder to regulate sample temperature. An aliquot of the compound in dioxane or DMSO is directly added to the desired solvent mixtures and mixed properly to achieve the required concentration and composition. For variable temperature absorption spectroscopy, freshly prepared aggregate solutions were heated and cooled at a rate of 1 K/min.

**Circular Dichroism (CD) Spectroscopy** was performed on Jasco J-815 CD Spectrophotometer. All optical spectroscopy-based experiments were carried out in a 1 mm path length quartz cuvette, using 300 µM solutions, unless mentioned otherwise. For variable temperature CD spectroscopy, freshly prepared aggregate solutions were heated and cooled at a rate of 1 K/min.

**FESEM** was performed on ZEISS SUPRA 55VP microscope. The sample was prepared by drop casting a solution of the aggregated compound on a glass coverslip. For nano-spherical aggregates, the drop-cast film was dried under static vacuum, whereas for secondary nanowire aggregates, the films were freeze-dried.

**Seed preparation protocol and seeding experiment details**: The Agg-MT solution was sonicated in a temperature-controlled sonicator for approximately 15 minutes at 20 °C. Subsequently, the resulting solution is employed as a seed solution for the seeding experiment. Seeds are added to the solution of Agg-P_K_. The net molecular concentration was maintained at 300 µM for various seed-to-monomer ratios.

**Simulation Models and Details**

We performed Molecular Dynamics simulations in the GROMACS software ^1^, version 20xx using the CHARMM36 force field ^2^. **L-PhePBI** was modeled using the Ligand Reader and Modeler ^3^ in the CHARMM-GUI server ^4^. For the solvents, DMSO parameters were taken from CGENFF ^5^, which is used for modeling small molecules, while the CHARMM-TIP3P model ^6^ was used for water. After building the initial configuration, the system is energy minimized using steepest descent method. This is followed by constant volume (NVT) equilibration and constant pressure (NPT) equilibration with position restraint on the heavy atom of **L-PhePBI**. Finally, the NPT production run is performed without any restraint on atoms. We use the V-rescale thermostat ^7^ for temperature coupling and C-rescale barostat ^8^ for pressure coupling. The LINCS algorithm ^9^ is applied to constrain hydrogen atom bonds. The Verlet cutoff scheme ^10^ is implemented with a 1.0 nm cutoff. Periodic boundary conditions are maintained in x, y and z directions.

**Simulations for Solvent Effect**

We randomly insert 100 molecules of **L-PhePBI** into an 11.3 nm cubic box. Next, we add DMSO and water molecules to the box at different volume/volume concentration ratios between the two solvents, i.e., neat DMSO, neat water, 4:6, and 6:4 (Figure S4a, S4b, and S4c). We perform NVT equilibration for 10 ns followed by NPT equilibration for 100 ns and production simulation for 1 𝜇s (Figure S5a, S5b, and S5c).

We also perform simulations of preformed assembly of **L-PhePBI** in the two mixture solvent systems (Figure S6a and S6b). For this, we stack 50 molecules of **L-PhePBI** in a cuboidal box with dimension 12 nm x 42 nm x 12 nm. The stacked assembly is constructed with a 40° angle (twist angle of the nearest neighbours gives the first peak at ~ 40° angle in the previous randomly inserted system) and 3.0 Å distance between consecutive molecules (Figure S4b and S4d). We then insert DMSO and water molecules according to the desired volume fraction. For the stacked assembly, the final NPT production run is conducted for 100 ns (Figure S6a and S6b). For both the set-up the temperature is maintained at 298 K, and the pressure is set to 1 bar.

**Simulations for Temperature Effect**

We study the case of the solvent composition ratio 6:4 for DMSO: water at two different temperatures, 298 K and 363 K at 1 bar pressure. Similar to the preformed assembly used in the previous case, we begin by stacking **L-PhePBI** molecules at 3.0 Å distance between consecutive molecules. Different initial configurations are constructed by varying the angles between consecutive molecules per configuration (2° to 40° and -10° to -40° with an interval of 2°). In total, we have 36 different initial configurations. For each configuration, we perform NVT equilibration for 2 ns, NPT equilibration for 2 ns, and NPT production for 10 ns.

**Twist Angle Analysis**

To calculate the angle between a pair of molecules in the assembly, the twist angle ^11^ is defined as,

$\theta_{1}=\sigma_{n}{cos}^{-1}(\hat{\nu}_{i}.\hat{\nu}_{j})$, where $\hat{\nu}_{n}$ is a unit vector defined as

$\hat{\nu}_{n}=\frac{\bar{L}_{n}}{\left| \bar{L}_{n} \right|}$, and $\bar{L}_{n}$ is the vector joining the two nitrogens of the **L-PhePBI** molecule (Figure S1). The value of $\sigma_{n}$ indicates the orientation of the molecules in the assembly relative to the positive y-axis, with:

$\sigma_{n}=+1$ determines the clockwise and $\sigma_{n}=-1$ determines the anticlockwise orientation.


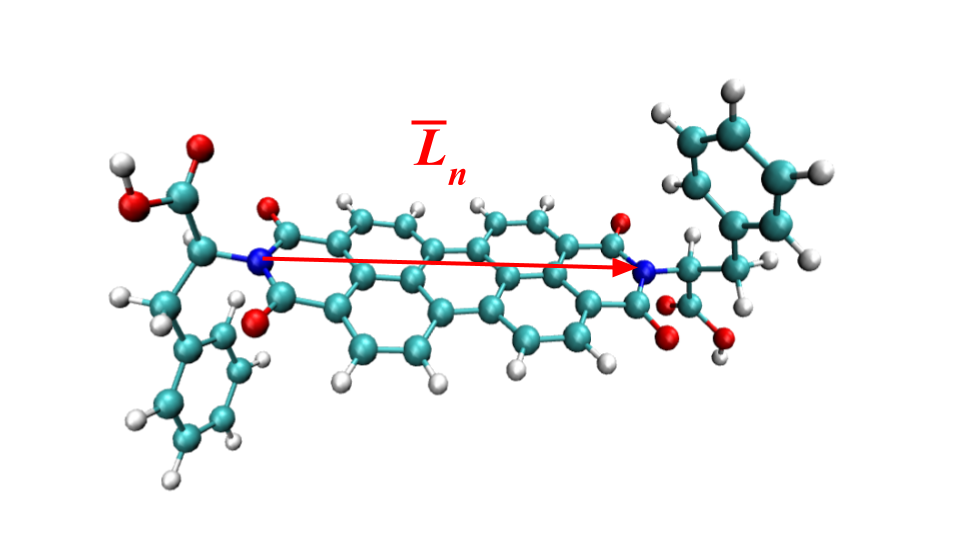


Figure S1. Red arrow represents the vector $\underline{L}_{n}$, joining the two nitrogen atoms of the imide groups.

**Orientational Tetrahedral Order Parameter (OTO)**

To study the effect of solvent on the assembly, we employ the widely used OTO parameter ^12^. It is defined as, $q=1-\frac{3}{8}\sum_{j=1}^{3} \sum_{k=j+1}^{4} \left( cos\psi_{jk}+\frac{1}{3} \right)^{2}$

where angle $\psi_{jk}$ is formed by the lines joining the oxygen of the water molecule, respectively, for which the OTO parameter is being calculated, and its nearest neighbor *j* and *k* oxygen atoms. Water molecules within a cutoff distance of 0.5 nm (first hydration shell) are considered to calculate OTO near the **L-PhePBI** assembly. Around 16 to 70 water molecules are found within the cutoff distance of 0.5 nm from the solute molecules.

**2. Synthesis and Characterization:**

Figure S2. Synthetic scheme of **L-PhePBI**

**L-PhePBI** was synthesized and characterized according to the procedure reported earlier.^13^

**3. Additional Information**

Figure S3. a) Composition-dependent CD spectra of **L-PhePBI**, conc. 300 μM. b) Composition-dependent optical absorption spectra, conc. 300 μM, 298 K. c) FESEM morphologies of **L-PhePBI** aggregates in water d) Plot of *E*_T_ (30) Polarity of DMSO-water mixture as a function of water volume fraction (*f*_water_) shows no drastic change around the critical compositions *f*_water_ = 0.4−0.6.

Figure S4. Composition-dependent CD spectra of **L-PhePBI**, conc. 300 μM a) Different DMF-water mixtures, b) Dioxane-water, c) Acetonitrile-water mixtures. Path length used 1 mm.


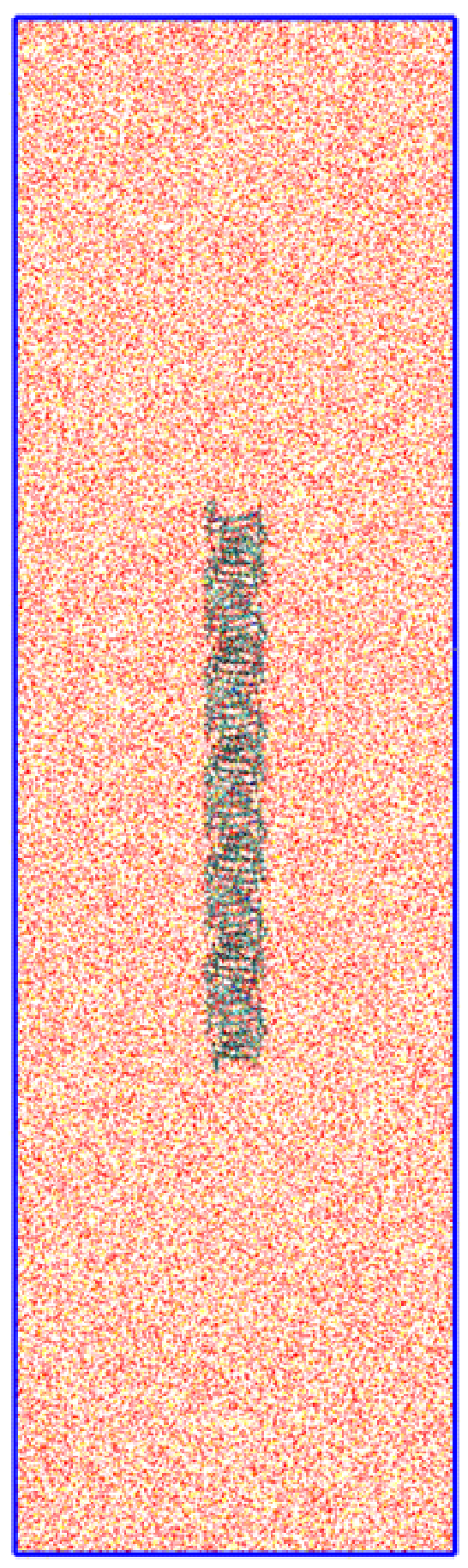

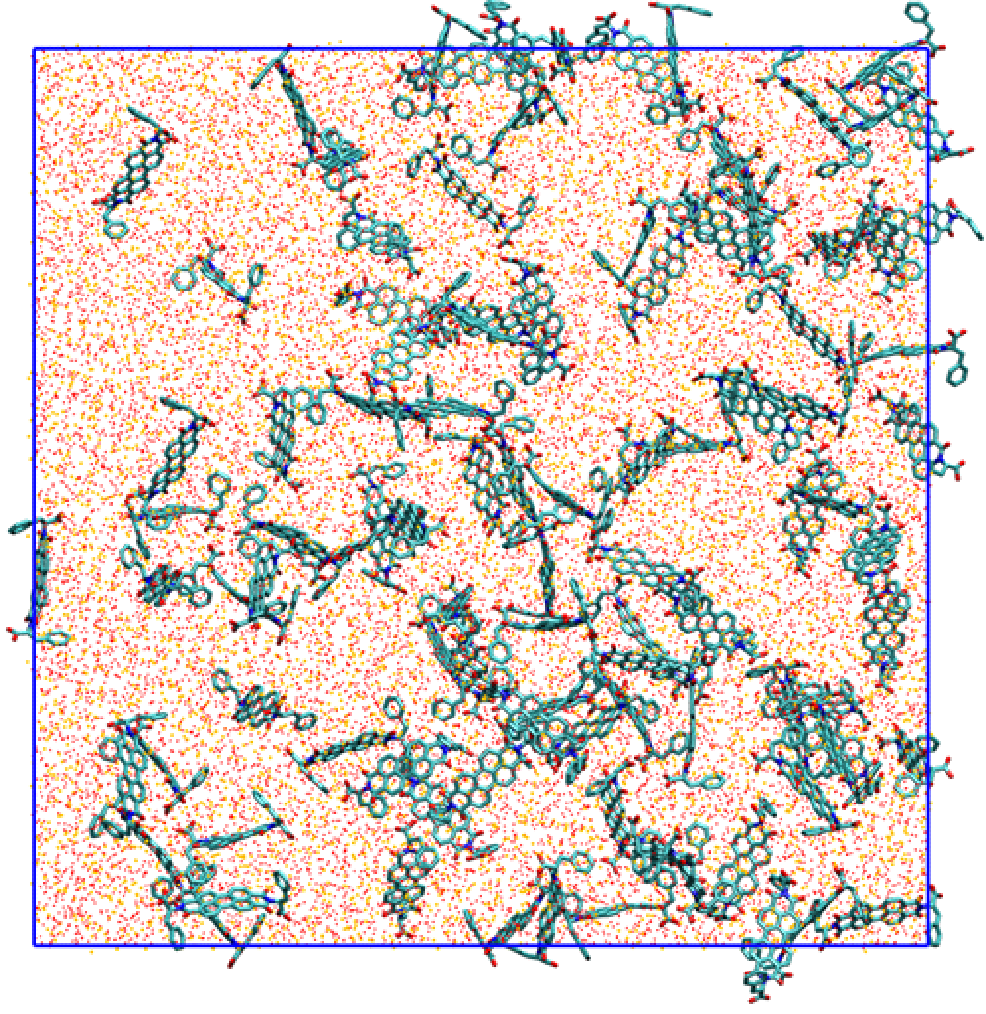

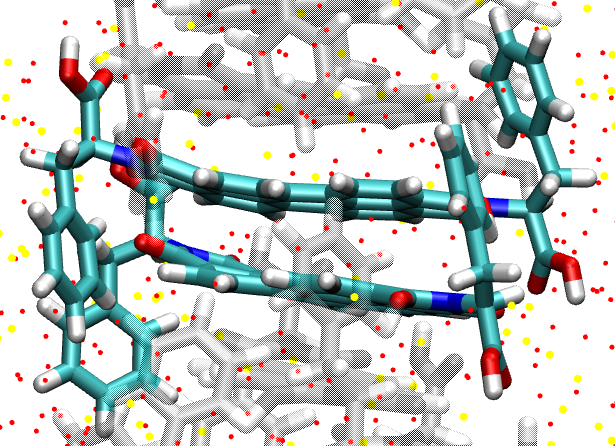


**a**

**b**

**c**

**
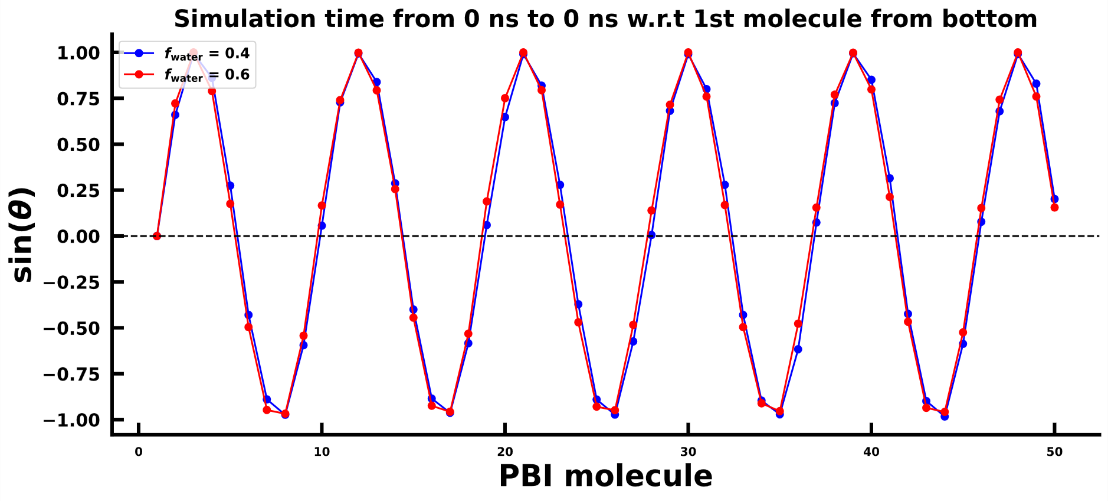
d**

Figure S5. MD simulation initial configurations. (a) Randomly inserted **L-PhePBI** system. (b) Preformed **L-PhePBI** assembly system. (c) Zoomed-in snapshot of preformed assembly. (d) Median plot of the sine of twist angles of preformed assembly molecules relative to the first molecule.

Figure S6. Final configuration snapshots after 1 𝜇s simulation of randomly inserted 100 **L-PhePBI** molecules in (a) neat water, (b) *f*_water_ = 0.4 and (c) *f*_water_ = 0.6 solutions. Water and DMSO molecules are not shown for clear visualization.

**a**
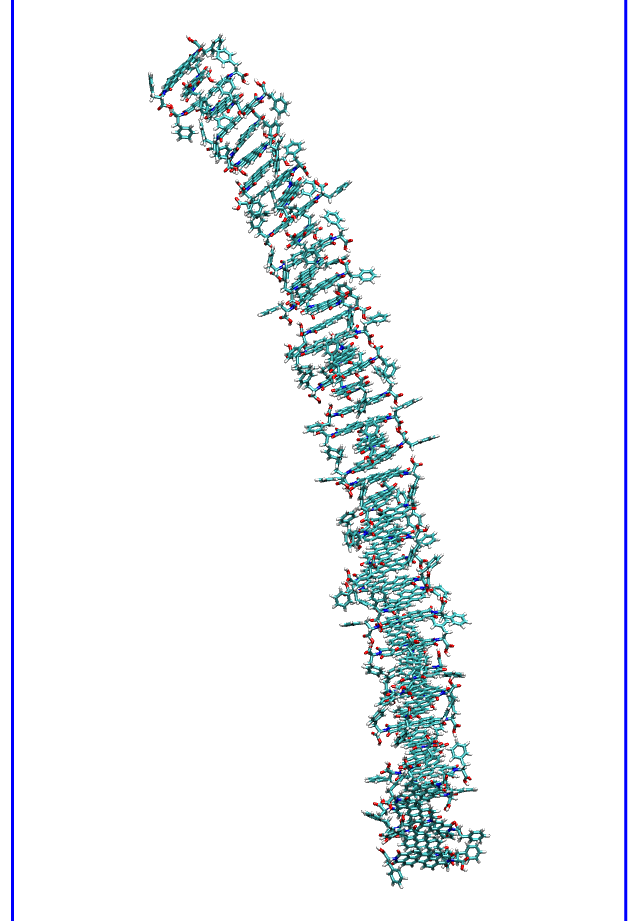
 **b**
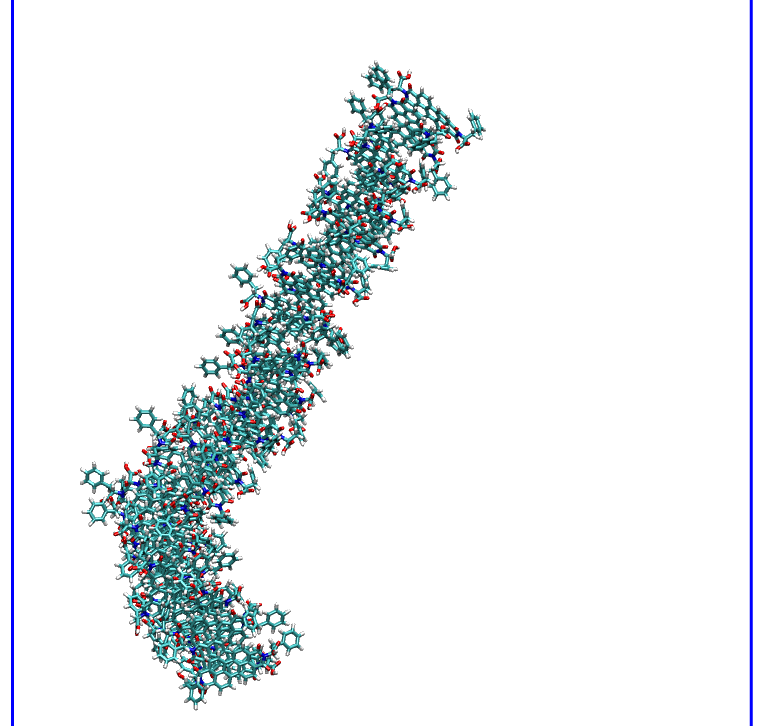


Figure S7. Zoomed-in final configuration snapshot of the preformed assembly after 100 ns simulation in a) water: DMSO = 4:6 and (b) water: DMSO = 6:4. Water and DMSO molecules are not shown for clear visualization.


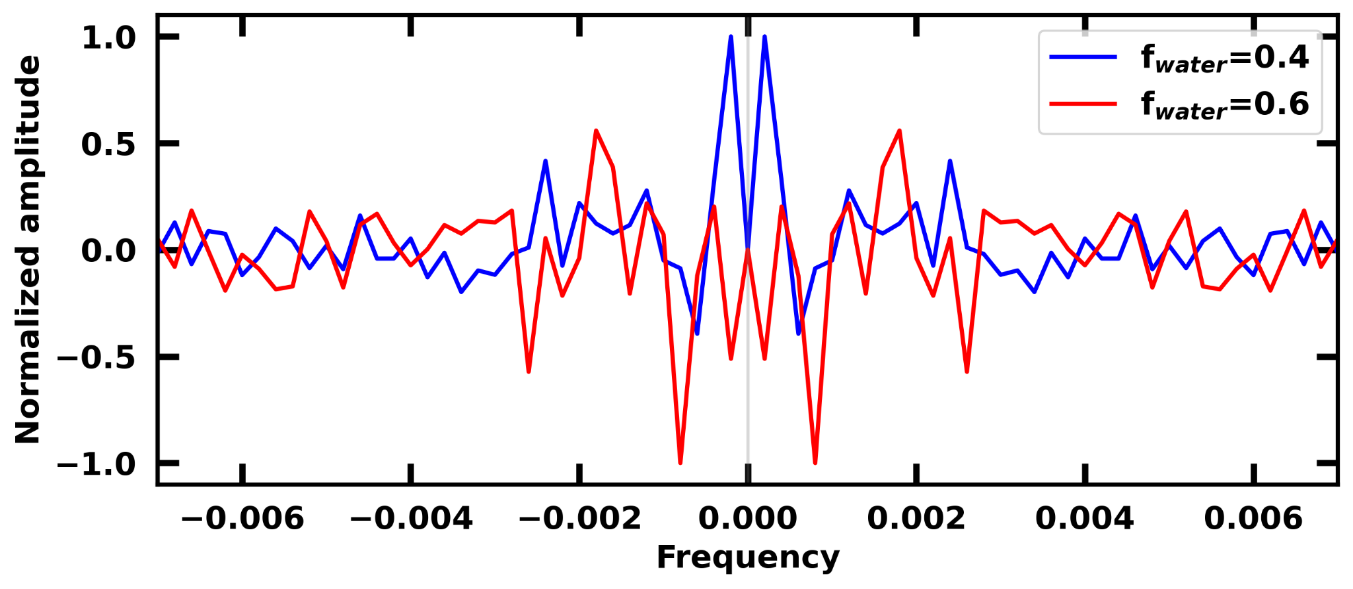


Figure S8. ((|Δsin(θ)| ≥ 0.5 from Figure 2b) over the 80–85 ns time window. The averaged spectra exhibit opposite signs for (*f*_water_ = 0.4) and (*f* _water_ = 0.6), indicating distinct solvent-dependent orientational ordering.


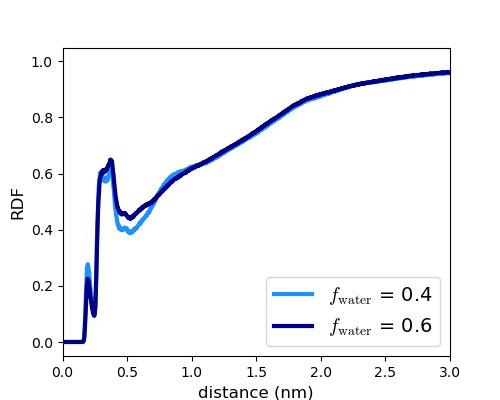


Figure S9. Radial distribution of water in the **L-PhePBI** assembly system in the two solvent compositions.

Figure S10. Visual representation of OTO values (a) negative (-0.26) and (b) positive (0.25).

**
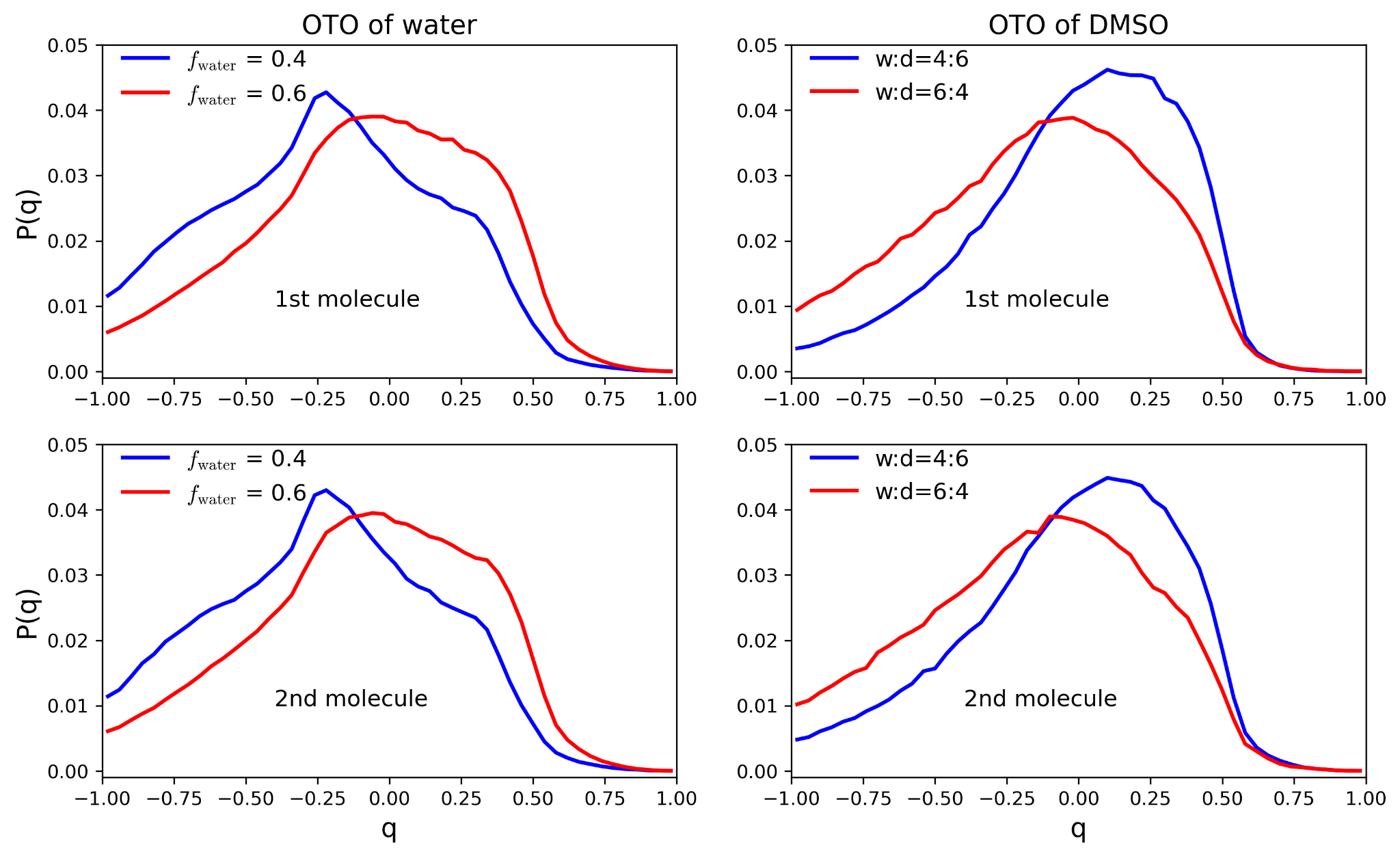
**

Figure S11. Orientational tetrahedral order (OTO) parameter of water in a dimer system around **L-PhePBI** molecules within 0.5 nm cutoff.

**Hydrogen bond calculation:**

Hydrogen bonds are calculated using the Hydrogen Bond Analysis module of the MD Analysis package. A hydrogen bond is defined using a donor-acceptor cutoff distance of 3.5 Å and a donor-hydrogen-acceptor angle of ≥ 150°. Continuous-time autocorrelation is determined for the identified hydrogen bonds, and the average lifetime is obtained by integrating the autocorrelation function over time.


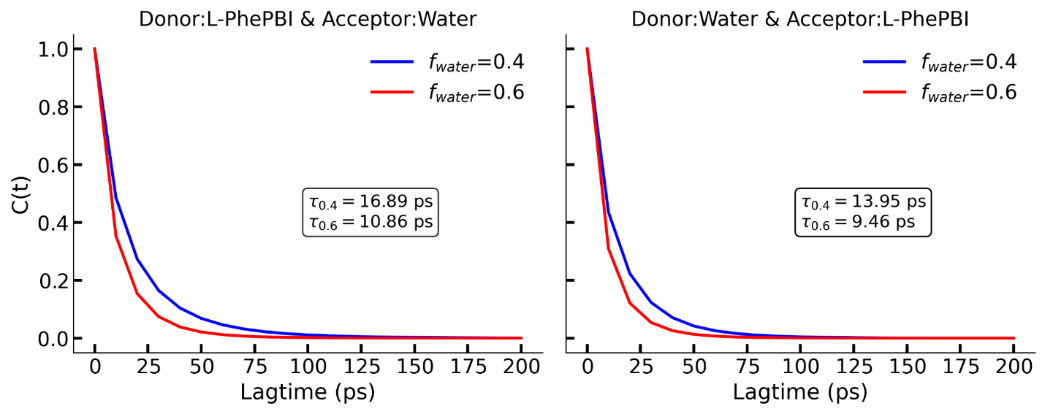


Figure S12: Continuous hydrogen-bond correlation functions, *C*(t) for hydrogen bonds formed between water and L-PhePBI at different solvent compositions. Left panel: L-PhePBI acts as the hydrogen-bond donor and water acts as the acceptor. Right panel: water acts as the hydrogen-bond donor and L-PhePBI acts as the acceptor. Average hydrogen-bond lifetimes (τ) obtained from the correlation functions are indicated in each panel. In both donor-acceptor configurations, the hydrogen-bond lifetime decreases with increasing water fraction, indicating faster solute-water hydrogen-bond dynamics at higher water content.

Figure S13. Variable temperature CD spectroscopy in *f*_water_ = 0.6 DMSO−water mixture, conc. 300 μM (a) heating run (b) cooling run. Variable temperature absorption spectra of **L-PhePBI** in *f*_water_ = 0.6 DMSO−water mixture, conc. 300 μM. (c) heating run, (d) cooling run. The heat-cool rate was maintained 1 K/min throughout all the experiments.

Figure S14. Agg-M_T_ formed by thermal stereomutation in *f*_water_ = 0.4 DMSO−water is subjected to a second thermal cycle. Upon (a) heating Agg-M_T_ from 293 K to 363 K, and (b) cooling it back to 293 K, no further aggregate reorganization or stereomutation is seen.

Figure S15. Variable temperature UV spectroscopy of **L-PhePBI** in *f*_water_ = 0.4 DMSO−water mixture (a) Heating run, (b) cooling run, (c) change in optical absorption of **L-PhePBI** aggregate in *f*_water_ = 0.4 DMSO−water mixture before and after thermal cycling. The heat-cool rate was maintained 1 K/min throughout all the experiments.

Figure S16.Variable temperature CD spectroscopy in *f*_water_ = 0.4 DMSO−water mixture during a) heating and b) cooling runs at 5 K/min. Faster heat-cool traps the kinetic Agg-P_K_ state and prevents its stereomutation into the more stable Agg-M_T_; conc. 300 μM.

Figure S17. (a) FESEM image of Agg-M_T_ at 298 K, and (b) Linear dichroism (LD) from **L-PhePBI** aggregate in different compositions has a very negligible contribution, comparable to that of monomeric **L-PhePBI** Conc. 300 μM.


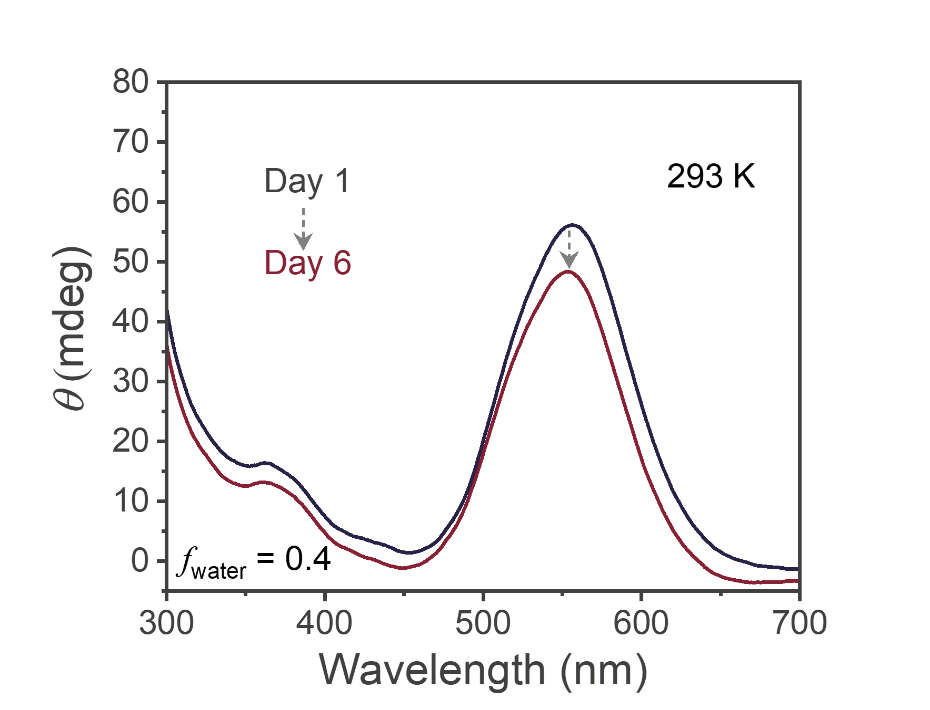


Figure S18. As-prepared Agg-P_K_ remains stable in solution up to one week.

**a
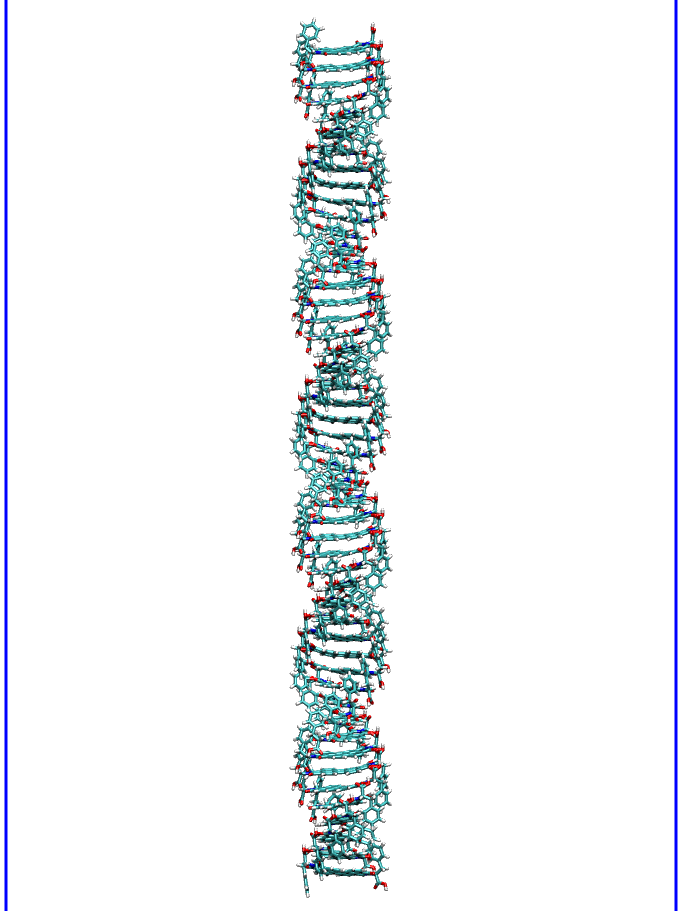
 b
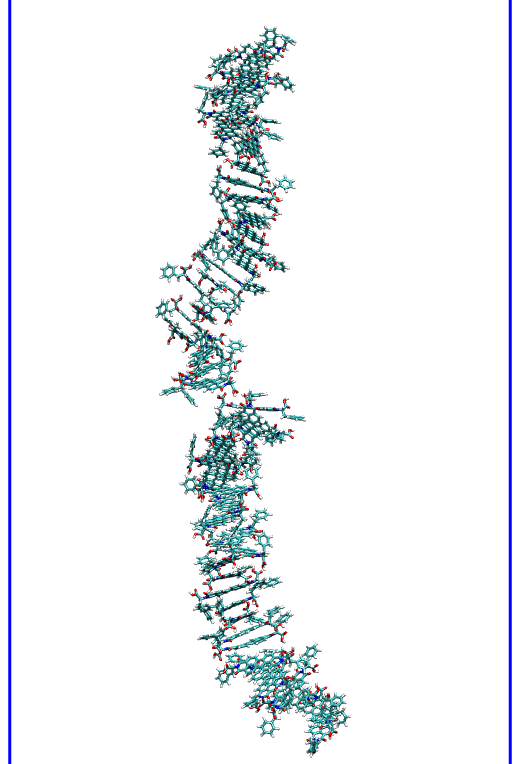
 c
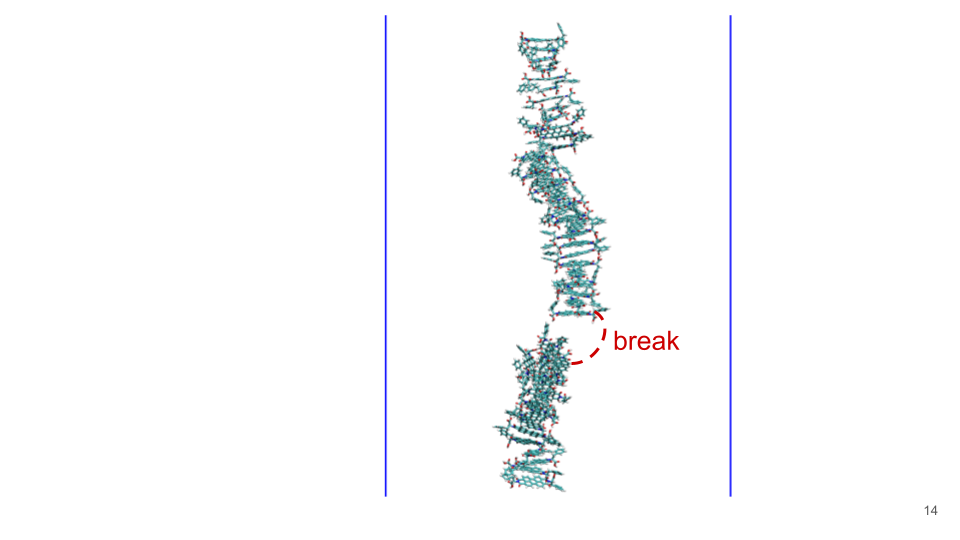
**

Figure S19. Snapshot of (a) initial frame (b) final frame at 298 K (c) final frame at 363 K after 10 ns simulation for an initial configuration. Red dotted curve indicates a break in the assembly. Water and DMSO molecules are not shown for clear visualization.

**a
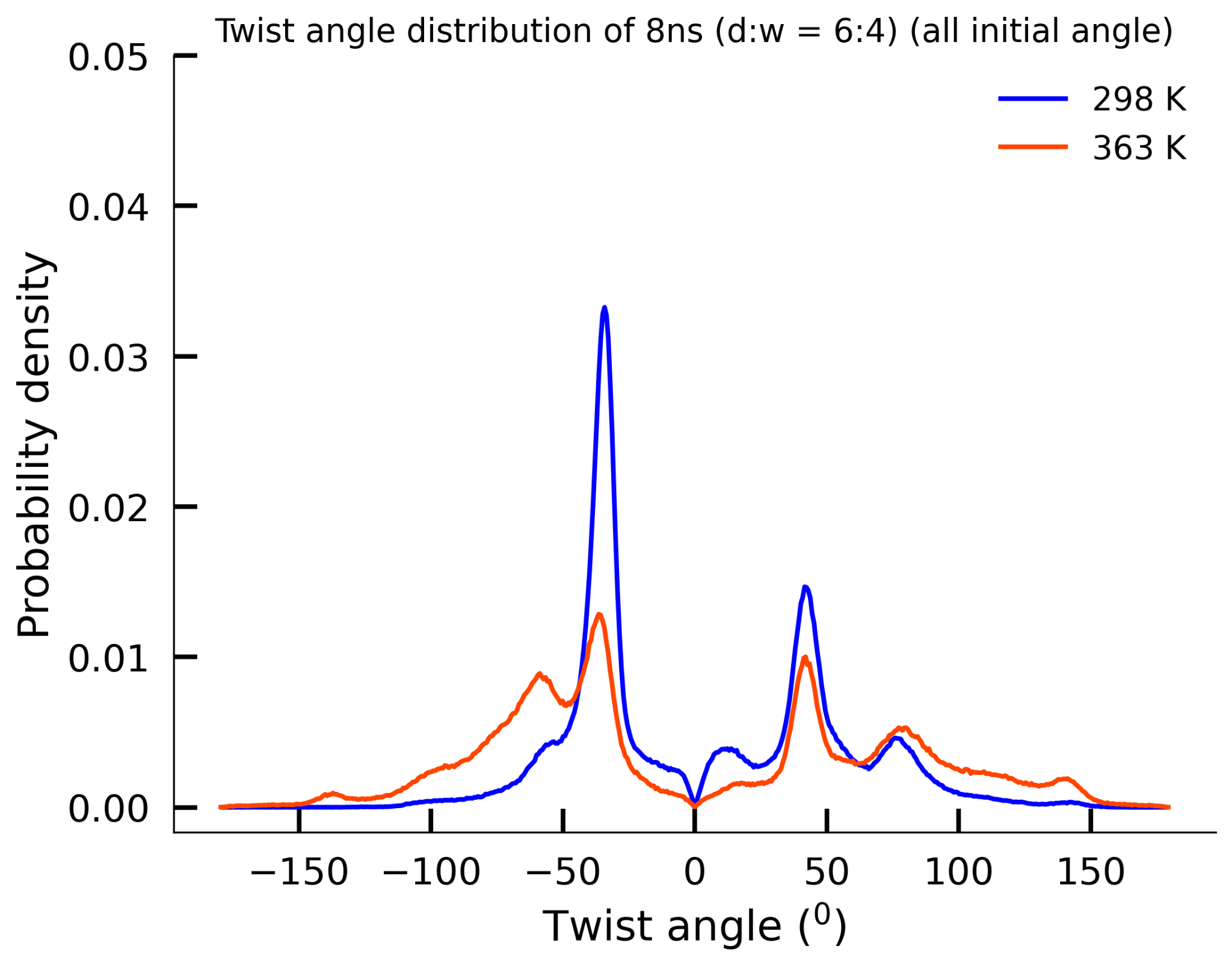
 b
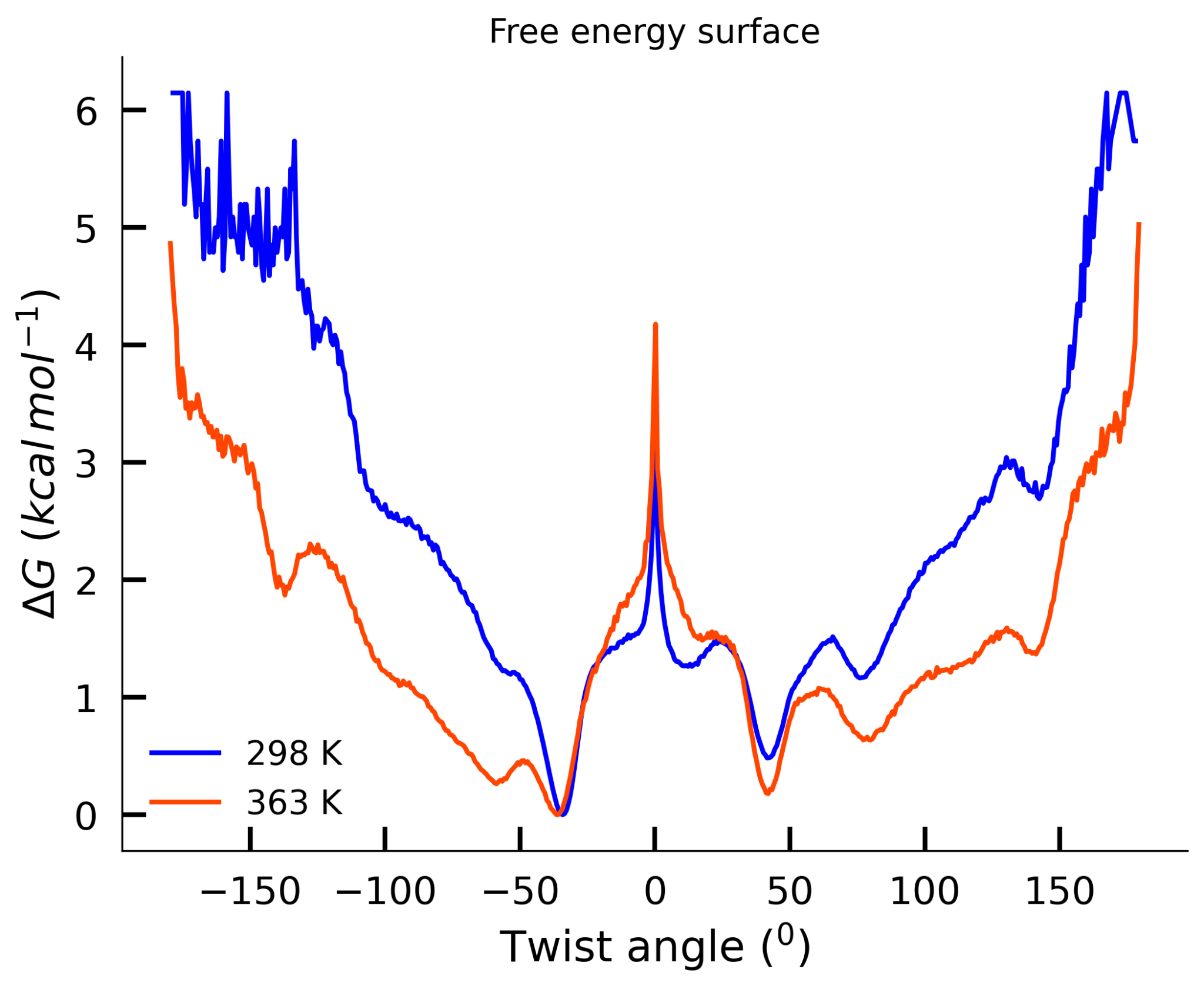
**

Figure S20. (a) Probability density distribution of twist angles at 298 K and 363 K, and (b) corresponding free energy surface. All the initial configurations are included.

**4. References**

1. Abraham, M.J., Murtola, T., Schulz, R., Páll, S., Smith, J.C., Hess, B. and Lindahl, E., 2015. GROMACS: High performance molecular simulations through multi-level parallelism from laptops to supercomputers. *SoftwareX*, *1*, pp.19-25. (<https://doi.org/10.1016/j.softx.2015.06.001>)
2. Huang, J. and MacKerell Jr, A.D., 2013. CHARMM36 all‐atom additive protein force field: Validation based on comparison to NMR data. *Journal of computational chemistry*, *34*(25), pp.2135-2145. (<https://doi.org/10.1002/jcc.23354>)
3. Kim, S., Lee, J., Jo, S., Brooks III, C.L., Lee, H.S. and Im, W., 2017. CHARMM‐GUI ligand reader and modeler for CHARMM force field generation of small molecules. (<https://doi.org/10.1002/jcc.24829>)
4. Jo, S., Kim, T., Iyer, V.G. and Im, W., 2008. CHARMM‐GUI: a web‐based graphical user interface for CHARMM. *Journal of computational chemistry*, *29*(11), pp.1859-1865. (<https://doi.org/10.1002/jcc.20945>)
5. Vanommeslaeghe, K., Hatcher, E., Acharya, C., Kundu, S., Zhong, S., Shim, J., Darian, E., Guvench, O., Lopes, P., Vorobyov, I. and Mackerell Jr, A.D., 2010. CHARMM general force field: A force field for drug‐like molecules compatible with the CHARMM all‐atom additive biological force fields. *Journal of computational chemistry*, *31*(4), pp.671-690. (<https://doi.org/10.1002/jcc.21367>)
6. MacKerell Jr, A.D., Bashford, D., Bellott, M.L.D.R., Dunbrack Jr, R.L., Evanseck, J.D., Field, M.J., Fischer, S., Gao, J., Guo, H., Ha, S. and Joseph-McCarthy, D., 1998. All-atom empirical potential for molecular modeling and dynamics studies of proteins. *The journal of physical chemistry B*, *102*(18), pp.3586-3616. (<https://doi.org/10.1021/jp973084f>)
7. Bussi, G., Donadio, D. and Parrinello, M., 2007. Canonical sampling through velocity rescaling. *The Journal of chemical physics*, *126*(1). (<https://doi.org/10.1063/1.2408420>)
8. Bernetti, M. and Bussi, G., 2020. Pressure control using stochastic cell rescaling. *The Journal of Chemical Physics*, *153*(11). (<https://doi.org/10.1063/5.0020514>)
9. Hess, B., Bekker, H., Berendsen, H.J. and Fraaije, J.G., 1997. LINCS: a linear constraint solver for molecular simulations. *Journal of computational chemistry*, *18*(12), pp.1463-1472. (<https://doi.org/10.1002/(SICI)1096-987X(199709)18:12%3C1463::AID-JCC4%3E3.0.CO;2-H>)
10. Grubmüller, H., Heller, H., Windemuth, A. and Schulten, K., 1991. Generalized Verlet algorithm for efficient molecular dynamics simulations with long-range interactions. *Molecular Simulation*, *6*(1-3), pp.121-142. (<https://doi.org/10.1080/08927029108022142>)
11. Yu, G. and Wilson, M.R., 2022. Molecular simulation studies of self-assembly for a chromonic perylene dye: All-atom studies and new approaches to coarse-graining. *Journal of Molecular Liquids*, *345*, p.118210. (<https://doi.org/10.1016/j.molliq.2021.118210>)
12. Duboué-Dijon, E. and Laage, D., 2015. Characterization of the local structure in liquid water by various order parameters. *The Journal of Physical Chemistry B*, *119*(26), pp.8406-8418. (<https://doi.org/10.1021/acs.jpcb.5b02936>)
13. Pal, T.; Chaudhuri, D. Chiral and Morphological Anisotropy of Supramolecular Polymers Shaped by a Singularity in Solvent Composition*. J. Am. Chem. Soc. 2023*, *145* (4), 2532–2543. https://doi.org/10.1021/jacs.2c12253.
